# An Overview of the Main Genetic, Epigenetic and Environmental Factors Involved in Autism Spectrum Disorder Focusing on Synaptic Activity

**DOI:** 10.3390/ijms21218290

**Published:** 2020-11-05

**Authors:** Elena Masini, Eleonora Loi, Ana Florencia Vega-Benedetti, Marinella Carta, Giuseppe Doneddu, Roberta Fadda, Patrizia Zavattari

**Affiliations:** 1 Department of Biomedical Sciences, Unit of Biology and Genetics, University of Cagliari, 09042 Cagliari, Italy; elena.masini97@gmail.com (E.M.); eleonora.loi@unica.it (E.L.); anaf.vegab@unica.it (A.F.V.-B.); 2 Center for Pervasive Developmental Disorders, Azienda Ospedaliera Brotzu, 09121 Cagliari, Italy; marinellacarta@aob.it; 3 Centro per l’Autismo e Disturbi correlati (CADc), Nuovo Centro Fisioterapico Sardo, 09131 Cagliari, Italy; io.donez@gmail.com; 4 Department of Pedagogy, Psychology, Philosophy, University of Cagliari, 09123 Cagliari, Italy; robfadda@unica.it

**Keywords:** autism spectrum disorder, ASD, genetic factors, epigenetic factors, environmental factors, pervasive developmental disorder, post-synaptic density, CNV, SNP, gene fusion

## Abstract

Autism spectrum disorder (ASD) is a neurodevelopmental disorder that affects social interaction and communication, with restricted interests, activity and behaviors. ASD is highly familial, indicating that genetic background strongly contributes to the development of this condition. However, only a fraction of the total number of genes thought to be associated with the condition have been discovered. Moreover, other factors may play an important role in ASD onset. In fact, it has been shown that parental conditions and in utero and perinatal factors may contribute to ASD etiology. More recently, epigenetic changes, including DNA methylation and micro RNA alterations, have been associated with ASD and proposed as potential biomarkers. This review aims to provide a summary of the literature regarding ASD candidate genes, mainly focusing on synapse formation and functionality and relevant epigenetic and environmental aspects acting in concert to determine ASD onset.

## 1. Introduction

Autism is a complex syndrome characterized by a range of conditions and symptoms that frame it as a spectrum of disorders (autism spectrum disorder, ASD), including relevant physiological and biochemical ones, whose core symptoms includes social deficits and restrictive/repetitive behaviors. In the 1960s, thanks to the studies of Bernard Rimland, it was understood that ASD is a psychiatric disorder which might be grounded on a combination of genetic and environmental factors. To cope with ASD, it is necessary to achieve positive results through multidisciplinary, biomedical and behavioral therapies. Early diagnosis and intensive therapeutic interventions greatly improve the disease outcomes. In this context, the discovery of new diagnostic methods to detect ASD-related genetic alterations and biomarkers becomes fundamental in order to make an early diagnosis of the disorder.

In this review, we provide an overview of the genetic, epigenetic and environmental factors contributing to ASD pathogenesis.

## 2. Autism

### 2.1. Clinical Characteristics of ASD

ASD can be considered as a group of early-onset neuroevolutionary disorders which seem to be at the basis of alterations in brain connectivity, with cascading effects on many neuropsychological functions [1,2]. The The Diagnostic and Statistical Manual of Mental Disorders (DSM-5) (The American Psychiatric Association, APA, Philadelphia, PA, USA, 2013) gives the condition of autism the attribute of “spectrum” and uses criteria derived from diagnostic research assessment tools.

As indicated in the DSM-5 (APA, Philadelphia, PA, USA, 2013), individuals with ASD are characterized by persistent deficits in social communication and social interaction across multiple contexts and by restricted, repetitive patterns of behaviour, interests or activities. Deficiency in social communication and social interaction might appear in the form of deficits in social-emotional reciprocity, in nonverbal communicative behaviours used for social interaction and deficit in developing, maintaining and understanding age-appropriate relationships. As reported in the DSM-5, symptoms could be masked during early development and fully manifest only when social demands exceed limited capacities or may be hidden by learned strategies in later life. The impairments should cause clinically significant damage in social, occupational or other important areas of current functioning.

ASD symptoms should not be better explained by a diagnosis of intellectual disability (ID) or global developmental delay. Intellectual disability and ASD might co-occur.

The DSM-5 proposes differentiations based on commorbidity with intellectual impairment, language impairment, another neurodevelopmental, mental, or behavioral disorder, genetic or medical condition or environmental factors. Furthermore, it is possible to differentiate between different levels of severity according to the level of support required to function in daily contexts. According to this description, it is clear that different clinical variants of ASD exist and should be taken into account for diagnosis and intervention (APA, 2013). A distinction is made between a congenital form of ASD, representing a small percentage of cases in which the symptoms occur shortly after birth and in which the genetic fingerprint is prevalent, and a regressive or acquired form, in which the disorder appears after a period of typical development and it is not characterised by typical and constant genetic abnormalities, although several single nucleotide polymorphisms (SNPs) have been associated with the disease [3,4]. SNPs constitute variations of a single nucleotide in certain DNA traits. SNPs associated with ASD have been identified in genes encoding for proteins involved in different processes, including: cellular detoxification, some neuronal receptors and metabolism of several neurotransmitters and metabolites, in particular those of the metabolic circuits of methylation and transulfuration [5,6].

### 2.2. Epidemiology

Autism has been considered relatively rare for many years, with a prevalence of less than 1 in 1000 children, while today, the estimated rate is 1 in 160, and it seems likely to increase in the coming years (World Health Organization, Geneva, Switzerland, 2019). In the last decade, the study of ASD genetics has proved to be crucial not only to interpret and explain its phenotypic heterogeneity but also to discover new diagnostic procedures and therapies. It is estimated to-date that hundreds of genes are involved in ASD, resulting in a unified spectrum of different phenotypes, including different language and social deficits with various associated sub-phenotypes [7].

ASD show an unequal distribution based on gender: males have a four times higher risk of developing the disorder than females. A number of hypotheses have been made to interpret this unequal prevalence: in females, a higher dose of genetic “defect” is required than in males, consistent with the hypothesis of the contribution of protective genetic factors in females, because in males, there is only one X chromosome, so when alterations appear, it cannot be compensated by the normality of a second X chromosome. An association between testosterone levels and ASD risk has also been described: males have more frequent inflammatory reactions and use the brain in a more focal way and therefore suffer more from alterations in neuronal development that affect the connection systems between the different areas [8].

Genetic, environmental and developmental factors play a key role in the onset of autism spectrum disorders, as highlighted from many epidemiological studies [9,10]. It is unlikely that a single condition or event plays a major role in the causality of ASD; based on research to date, rather, none of the risk factors identified is a necessary and sufficient condition for ASD. Even for syndromic or secondary autism, which refers to autism with a single defined cause, such as fragile X syndrome and tuberous sclerosis, none of these etiologies are specific to autism because each of them encompasses a variable proportion of individuals with and without autism [11]. At present, ASD appears to have a multifactorial etiology to which developmental (in utero and early childhood), environmental and genetic aspects contribute, in as-yet unknown and different ways. Emerging methodologies in genomics and epigenomics research could be the key to elucidate the mysteries underlying the epidemiology of autism spectrum disorder.

## 3. Genetic and Epigenetic Factors

The involvement of genetic etiology in ASD was first suggested by a study on twins reported in the 1970s. The genetic heritability of a trait can be estimated by comparing the phenotypic concordance between monozygotic twins (MZ), which have 100% genetic similarity, and dizygotic twins (DZ), who have approximately 50% genetic similarity. The greater the difference between the concordance of MZ twins and DZ twins, the higher the genetic heritability and the contribution of genetics to that trait. The genetic fingerprint was confirmed by the high concordance of autism in monozygotic twins (60–90%) compared to dizygotic twins (5–40%) [12,13].

One of the largest studies to date, involving more than two million children born between 1982 and 2006 in Sweden, concluded that ASD has an inheritability of 45–56% [14]. A study of all twins born in the UK between 1994 and 1996 estimated an inheritability of more than 56% using concordance in MZ (0.77–0.99) compared to DZ (0.22–0.65) twins [13].

Considering all lines of evidence, the genetic heritability of ASD is estimated to play a fundamental role in ASD onset, together with environmental and epigenetic factors. However, despite most of the studies aimed at understanding the etiological basis of ASD having been focused on the genetic component, it has been possible to associate genetic variants to only a relatively small fraction of ASD patients. The problem, known as the “missing heritability issue”, is common to most complex genetic diseases. Several hypotheses have been put forward to justify the missing heritability, such as the existence of poorly characterised variants, genotype/genotype interactions, incomplete penetrance, epigenetic factors and genotype/environment interactions [15,16,17].

In the 1990s, most of the research consisted of candidate gene studies, focusing on a particular gene that might be involved in ASD. From 2005, technologies such as whole-exome sequencing (WES), but also microarrays, have allowed genome-wide studies, leading to the identification of different variations in copy number (CNVs), DNA segments larger than 1 kilobase present in a variable copy number compared to a reference genome and single nucleotide variations (SNVs) in autistic patients, suggesting a highly heterogeneous genetic architecture. CNVs would contribute at about 15% and SNVs at 7% to the causes of ASD. Only a few of these genetic alterations have such complete penetrance that they are associated with ASD in almost every person who carries that variant [18]. On the contrary, genetic alterations with incomplete penetrance, variable expressivity or both are more frequently observed. However, the cause in most ASDs (>75%) remains elusive [18]. Genome-wide association studies (GWAS) have identified several SNPs associated with ASD. The first GWAS study allowed the identification of six polymorphisms, including some localized in *CDH10* and *CDH9*, genes encoding for cadherins, proteins that are important in cell adhesion, as common genetic variants in ASD [19]. However, the fact that several GWASs failed to identify some relevant loci despite the use of genetic data from more than 1000 families affected by autism suggests that the effect of individual common variants is relatively small [20].

On the other hand, WES analysis of affected and unaffected individuals has proven to be a powerful approach that offers new opportunities of sporadic cases studies and has the ability to detect mutations and de novo variants with incomplete penetrance [21]. WES has already led to the identification of over 150 new candidate genes for ASD.

In addition to SNPs, evidence is accumulating that CNVs play an important role in human neuropsychiatric diseases. ASD patients have three to five times more de novo CNVs than other family members and unaffected controls [22,23].

CNVs can influence gene expression, thus contributing to the pathogenesis of the disease through various mechanisms, including gene dosage, gene interruption, position effects, gene fusion and unmasking of recessive alleles or polymorphisms [24].

Screening for CNVs has proven to be a method of choice for identifying genes associated with ASD susceptibility [25]. Although CNVs associated with the disease are usually unique and show a low frequency in the population, they are identified in 8–21% of individuals with ASD and are most likely related to a severe clinical picture [26,27]. In addition, previous studies have indicated that individuals with syndromic ASD and intellectual disability have more pathogenic CNVs than individuals with non-syndromic ASD or ID [26,28].

CNVs may also lead to the generation of chimeric genes. Several studies have investigated whether fusion transcripts may lead to an increased ASD susceptibility. Holt and colleagues identified a fusion transcript involving *MAPKAPK5* and *ACAD10* genes in two ASD probands. However, the fusion transcript was detected at similar rates in both ASD patients and controls and had a premature stop codon, suggesting that it may be degraded by nonsense-mediated decay [29]. Similarly, Pagnamenta et al. identified a *DOCK4*-*IMMP2L* fusion transcript, likely to be subjected to nonsense-mediated decay in ASD individuals and their unaffected family members [30]. A study conducted on a multiplex family identified a *BST1*-*CD38* fusion transcript in one ASD proband with asthma, suggesting that it may be related to the more severe phenotype of this patient compared to the other ASD sibling [31]. qRT-PCR analysis showed that the fusion transcript was less expressed compared to the wild-type *BST1* transcript in the lymphoblastoid cell line derived from the proband, while the aberrant protein was not detected in a preliminary Western blot analysis [31]. Recently, our research group identified a microdeletion leading to the formation of an *ELMOD3*-*SH2D6* chimeric transcript in a multiplex ASD family [32]. *SH2D6* is expressed at extremely low levels in blood cells. On the other hand, the fusion transcript was highly expressed in PMBCs from the two ASD siblings and their unaffected mother carrying the deletion, suggesting that it was not subjected to nonsense-mediated decay. Bioinformatic analysis has shown that the fusion transcript would encode for a chimeric protein with an interrupted domain of ELMOD3 and would not contain the canonical SH2D6 sequence, suggesting an impaired function of the protein [32]. These results suggest a possible contribution of fusion transcripts in the complex ASD phenotype. Therefore, in case of copy number loss, the possible transcript fusion and chimeric protein product should be deeply investigated. 

Recent studies have shown that epigenetic factors, including DNA methylation, hystone modifications and microRNAs (miRNAs), could play an important role in predisposition to autism.

**Figure 1:**
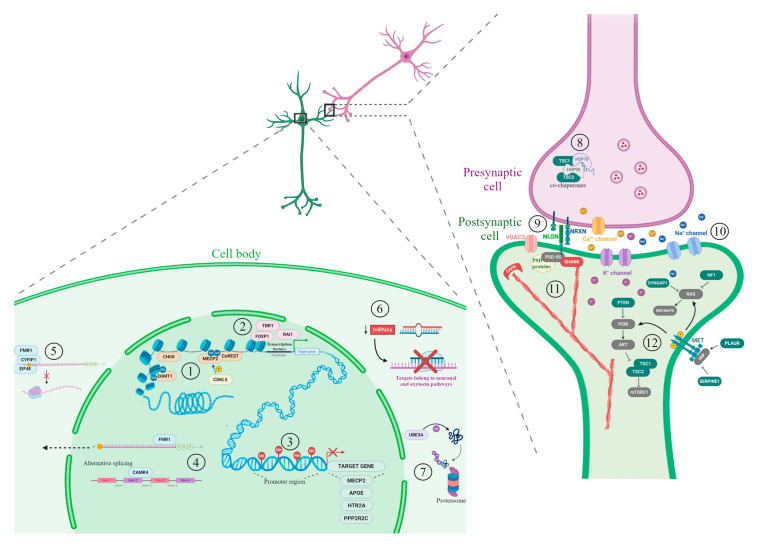
Candidate genes and epigenetic factors representative of the main processes involved in autism spectrum disorder (ASD) development. The illustration shows a synapse between neurons (presynaptic cell in violet and postsynaptic cell in green). On the bottom-left, a cell body of a neuron including different nuclear and cytoplasmic mechanisms involved in ASD. In the nucleus, several processes implicated in gene expression regulation are shown: (**1**) chromatin packaging and factors involved in chromatin remodeling; (**2**) gene transcription regulated by transcription factors; (**3**) DNA methylation at promoter region associated with transcription inhibition of target genes; (**4**) alternative splicing and mRNA export to the cytoplasm. In the cytoplasm, the following mechanisms are shown: (**5**) regulation of protein translation by the CYFIP1-EIF4E-FMR1 complex; (**6**) post-transcriptional regulation by miRNA; (**7**) protein ubiquitination and degradation by proteasome. On the right, the synapse architecture and functionality mechanisms associated with ASD. In the presynaptic cell, (**8**) TSC proteins and co-chaperons. (**9**) The neurexin/neuroligin transsynaptic complex and (**10**) the voltage-gated ion channels are represented. In the postsynaptic cell, (**11**) actin filaments, capping proteins and scaffold proteins; (**12**) some members of PI3K/AKT pathway, RAS signal transduction pathway and MET receptor tyrosine kinase pathway. Chromatin remodelers are indicated in beige, transcription factors in pink, proteins involved in RNA binding and export in light blue, protein ubiquitination in purple, scaffold proteins in red, cell growth and proliferation proteins in green and their related pathway members in grey. A more comprehensive list of ASD candidate genes can be found in Table 1 and along the text. Figure created using BioRender.com images.

We herein provide an overview of the main candidate genes (extensively reviewed in [33]) and epigenetic mechanisms involved in ASD etiology. Figure 1 summarizes some ASD candidate genes and epigenetic factors, belonging to the pathways mainly associated with ASD described in this review.

### 3.1. Relevant Candidate Genes

Case-control studies on population and animal models have pointed out more than 800 genes associated with autism. The most affected genes in ASD encode for proteins involved in chromatin remodeling and transcriptional regulation, cell proliferation and mostly synaptic architecture and functionality. In this review, we will focus on this last category since our recent studies have also pointed out alterations in these genes fundamental for a proper synaptic function. Table 1 provides a summary of several genes clearly implicated in ASD, included in the SFARI Gene database as high confidence ASD genes (release 26 October 2020, gene.sfari.org) belonging to the other categories. Most of these genes were indicated in the largest exome sequencing study of ASD to date [34], as well as in the list narrowing down the number of amygdala-expressed genes associated to the social pathophysiology of ASD [35].

#### Synaptic Architecture and Functionality

It is not surprising that many candidate ASD genes are involved in synaptic architecture and function, which allows the transmission of information between neurons and between neurons and other cells, such as muscle, sensory and other cells. Many ASD candidate genes are involved in dendritic spine formation. Dendritic spines are small actin-rich protrusions that form the postsynaptic part of most excitatory synapses. Remodeling of actin cytoskeleton is responsible for the changes in the shape and size of dendritic spines and, consequently, to the synaptic functions [36]. Actin regulation mechanisms regulate the formation, maturation and plasticity of dendritic spines and of neuronal processes, such as learning and memory [36]. Abnormalities in the number and shape of dendritic spines have been observed in several neurological disorders, including autism, and contribute to brain dysfunction [37].

Post-synaptic density proteins (PSD), including cell adhesion molecules, scaffold proteins, receptors and cytoskeleton proteins, are fundamental for synaptic transmission and plasticity. Alterations of these proteins have been associated with many neurological disorders, including ASD [38].

- Cell adhesion molecules

Neurexins (NRXN) and neuroligins (NLGN) are transmembrane synaptic proteins that form the neurexin/neuroligin transsynaptic complex, crucial for synaptic function [39]. NLGNs bind to SHANK3 through PSD-95 and other synaptic proteins.

Loss of function NRX1 variants in ASD individuals have been identified in multiple studies [23,40,41]. Studies conducted in animal models knockout (KO) for *NLGN* and *NRXN* family members showed that mice develop ASD-like symptoms and have confirmed their role in synaptic function [42,43,44].

*CNTNAP2*, also known as *CASPR2*, encodes for a member of the NRXN family that serves as an adhesion protein, primarily between neuronal and glial cells. CNVs encompassing *CNTNAP2* and resulting in its decreased expression have been described in subjects with ASD [45,46,47]. The suppression of CNTNAP2 in murine models causes autistic behaviors, such as repetitive behaviors and reduced socialization and communication [48].

- Scaffold proteins

SHANK gene family, including *SHANK1*, *SHANK2* and *SHANK3*, has been suggested as a strong candidate for ASD. SHANK proteins are multi-domain post synaptic density scaffold proteins that connect neurotransmitter receptors, ion channels and other membrane proteins to cytoskeleton actin and signaling proteins. These proteins are important for synapse formation and dendritic spine maturation [49]. Rare deletions of *SHANK2* and de novo variants causing loss of protein function have been identified in individuals with ASD [50]. A microdeletion encompassing *SHANK3* determines Phelan–McDermid syndrome, characterized by intellectual disability, ASD, severe speech disorders and epilepsy [51]. A meta-analysis of SHANK mutations found low frequency of *SHANK1* and *SHANK2* deleterious mutations in contrast to the high frequency of loss of function *SHANK3* mutations in cases with ASD [52].

Our studies led to the identification of another promising ASD candidate gene, *CAPG*. This gene encodes for a member of the gelsolin family of actin-regulatory proteins, important for the remodeling of actin architecture. A microdeletion encompassing the entire *CAPG* gene has been recently described in three completely independent families in the heterozygous [53,54] and homozygous state [55]. Importantly, a reduced CAPG expression, both at transcriptional and protein levels, has been detected in the Sardinian family members carrying the deletion, and reduced *CAPG* mRNA levels have been also observed in an independent cohort of 13 non-Sardinian ASD cases compared to age-matched healthy controls [54].

Several studies have demonstrated the importance of CAPG for the formation of functional synapses. In fact, experiments conducted on cultured hippocampal neurons have demonstrated that capping proteins are present at the branched actin filament network of dendritic spine heads and they are fundamental for dendritic spine development [56,57]. In fact, *CAPG* knock-down led to a decline in spine density and to an increased number of filopodia-like protrusions [56].

- Voltage-gated ion channels

The role of genetic defects of different ion channels in the pathogenesis of ASD is well established. In fact, GWAS, WES and WGS have identified several polymorphisms and rare variants in calcium, sodium and potassium channels in ASD subjects (reviewed in [58]).

Point mutations in *CACNA1C* gene, which encode for l-type voltage-gated Ca^2+^ channel Cav1.2, lead to Timothy syndrome (TS), a disorder affecting multiple organs and characterized by an autistic phenotype [59,60]. l-type channels are mainly expressed in neuronal dendrites and cell bodies and are crucial for the activation of Ca^2+^-signaling pathways and for neuronal excitability [61]. Defects in CACNA1C prevent the inactivation of the channel and lead to its prolonged opening and consequent increase in Ca^2+^ flux [60,62].

Mutations in other genes encoding for l-type and T-type Ca^2+^ channels, such as *CACNA1D*, *CACNA1E*, *CACNA1F* and *CACNA1H*, have been described in ASD [63,64,65,66,67]. Moreover, mutations of *CACNB2*, the gene encoding for the regulatory β2 subunit of CACNA1C, have been found in ASD families [68].

Genetic defects in genes encoding for sodium channels, such as *SCN1A*, *SCN2A*, *SCN3A*, *SCN7A* and *SNC8A*, have been also associated with ASD [69,70,71,72,73]. Voltage-dependent sodium channels are mainly expressed in neurons and glial cells and are fundamental for the initiation and propagation of action potentials. Mutations of SCN1A cause Dravet syndrome, characterized by seizures and frequently manifesting, also, autistic symptoms. A study has shown that *Scn1a*+/− heterozygous KO mice display stereotypical and anxious behaviors other than seizures [74].

Several studies have also shown that mutations in genes encoding for K^+^ channels, including *KCNMA1*, *KCND2*, *KCNJ10*, *KCNQ3* and *KCNQ5*, may play a central role in ASD etiology [75,76,77,78]. It has been shown that KO of *Fmr1* in mice results in K^+^ channel dysregulation and consequent dysregulation of synaptic transmission [79].

Some evidence has shown that voltage-dependent anion channel (VDAC) genes, a class of postsynaptic density genes highly expressed in several brain regions, could be implicated in ASD. In fact, autoantibodies against VDAC proteins have been detected in autistic individuals, suggesting a possible causal role in ASD pathogenesis [80]. Moreover, the beneficial effects observed in ASD patients treated with coenzyme Q, or other agents influencing the transport of electrons, have been attributed to the control of such molecules on the porin channels [81]. Recently, a study conducted by our research group identified a 2-bp frameshift deletion of *VDAC3* in an ASD family [54].

### 3.2. Epigenetic Factors

There is increasing evidence supporting the possible role of epigenetic aberrations, including DNA methylation alterations and microRNAs, in ASD etiology.

- DNA methylation

Several studies have conducted global methylation analyses in peripheral tissues as well as post-mortem brain tissues from ASD subjects and controls.

Studies conducted on lymphoblastoid cell lines and whole-blood DNA from monozygotic twins discordant for ASD diagnosis and controls identified several differentially methylated regions (DMRs) between discordant MZ twins and between ASD patients and control samples [82,83].

Zhu et al. identified 400 DMRs, enriched at promoters of genes involved in neuronal development, between placentas from children later diagnosed with ASD and those from typically developing controls. Methylation levels of two DMRs, mapping on *CYP2E1* and *IRS2*, were respectively associated with genotype within the DMR and prenatal vitamin use [84].

Conversely, a large epigenome-wide association study, performed on blood-DNA from 796 ASD cases and 858 controls, did not detect any differentially methylated CpG site after correction for multiple testing [85].

Similarly, Siu et al. did not detect any DNA methylation patterns clearly distinguishing heterogenous ASD cases from controls. However, they identified unique DNA methylation signatures for ASD individuals with 16p11.2 deletions or pathogenic variants of *CHD8* [86].

On the other hand, Kimura et al. identified a potential biomarker for adult ASD. The identified CpG site was hypermethylated in whole-blood DNA from ASD patients compared to controls and mapped on the *PPP2R2C* gene, which resulted down-regulated in ASD subjects [87].

Alterations in Alu methylation patterns have been observed in ASD cases sub-grouped based on Autism Diagnostic Interview-Revised scores compared with matched controls [88].

More recently, a genome-wide methylation study was performed on post-mortem tissue samples from different brain regions dissected from ASD subjects and controls. Wide-spread methylation differences, with more pronounced effects in cortical regions compared to cerebellum, were detected between idiopathic ASD cases and controls and in individuals carrying 15q11–13 duplication [89].

Another global methylation study has recently reported that differentially methylated CpG sites identified in ASD cases compared to controls are enriched in pathways converging on mitochondrial metabolism and protein ubiquitination, suggesting a possible role of DNA methylation and mitochondrial dysfunction in ASD [90].

Other studies measuring methylation levels of candidate genes using targeted approaches detected hypermethylation of genes including *APOE* [91] and *HTR2A* [92] and hypomethylation of genes such as *HTR4* [93] in ASD subjects compared to controls.

The functional impact of locus-specific *Mecp2* methylation on ASD onset has been recently demonstrated in vitro and in vivo. *MECP2* is known to be hypermethylated and down-regulated in ASD subjects. The authors of this study employed the CRISPR-dCas9 methylation editing system to induce methylation of the *Mecp2* transcription start site in Neuro-2a cells and in mouse models, resulting in *Mecp2* down-regulation and the acquisition of behavioral changes attributable to an ASD phenotype in mice [94].

- miRNA

Several studies have found that miRNA expression profiles are dysregulated in different matrices, including saliva, blood and brain tissues, from ASD individuals [95].

A study conducted on saliva samples from individuals with and without ASD has identified a panel of four microRNAs differentially expressed between ASD patients and controls [96]. A panel of five salivary miRNAs has shown an accuracy of about 90% in the detection of developmental disorders, including ASD [97].

Down-regulation of miR-6126 has been detected in peripheral blood samples from adult individuals with ASD. The predicted targets of this miRNA belong to neuronal and oxytocin pathways [98]. Similarly, Ozkul et al. identified a consistent decrease and a slight reduction in six microRNAs (miR-19a-3p, miR-361-5p, miR-3613-3p, miR-150-5p, miR-126-3p and miR-499a-5p) in serum samples from ASD children and their unaffected family members compared to healthy controls. This result was replicated in the blood, hypothalamus and sperm of two ASD mouse models [99]. Another study conducted on serum samples identified a panel of three miRNAs (miR-130-3p, miR-181b-5p and miR-320a) showing an area under the curve >0.85 in distinguishing ASD subjects from controls [100].

Studies conducted on knockout (KO) mouse models for some ASD candidate genes, including *Fmr1*, *Mecp2* and *Ube3A*, have observed dysregulation of different miRNAs and evaluated their regulatory role in neuronal context [101,102,103].

## 4. Environmental Factors

As mentioned above, several studies investigated the possible role of environmental factors in the etiology of ASD. According to recent studies, up to 40–50% of variance in ASD liability could be determined by environmental factors, such as drugs, toxic exposures, parental age, nutrition, fetal environment and many others [104,105,106]. However, while for some potential risk factors, there is strong evidence, supported by association studies but also by in vitro and in vivo studies, only weak associations have been described for many others.

**Figure 2:**
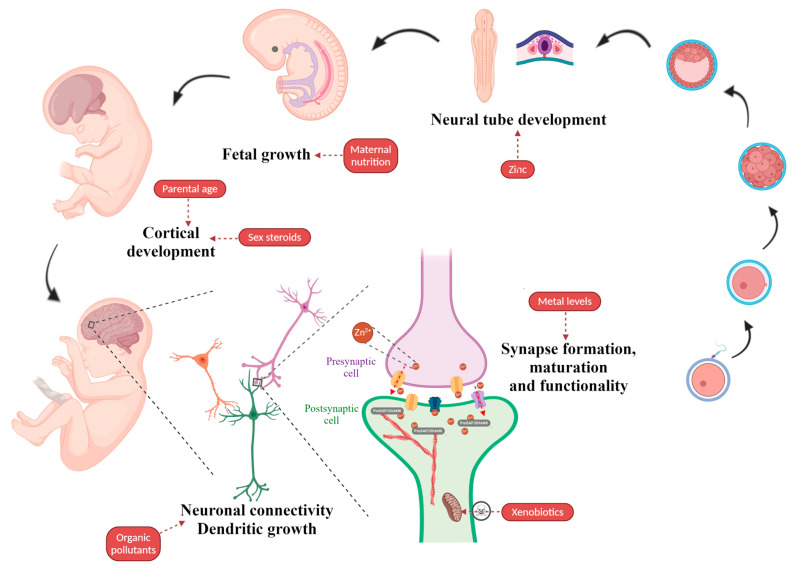
Environmental factors associated with ASD. The illustration indicates the putative impact of environmental factors on embryonic and fetal development with a particular focus on neuronal development and synaptic function. Figure created using BioRender.com images.

Below is reported an overview of the most-studied environmental factors that have been found to potentially contribute to cause ASD (recently reviewed in [107]) (Figure 2).

Parental age is one of the most established environmental ASD risk factors. In fact, much evidence has correlated advanced paternal age (APA) with the development of bipolar disorder, schizophrenia, ADHD and ASD [108]. A meta-analysis of 27 studies on the association between advanced parental age and ASD showed that a 10-year increase in maternal and paternal age is associated with a 20% higher risk of ASD in children [109]. A study has shown that age-related methylation changes observed in sperm could be related to an increased ASD risk in the offspring [110]. APA has been associated with reduced cortical thickness of the right posterior ventral cingulate cortex in ASD offspring [111]. Experiments conducted in mouse models have confirmed that APA is associated with the development of autism-like symptoms in the offspring [112,113] and with altered cortical morphology in male APA mice [112]. Moreover, behaviors related to ASD have also been observed in the second generation of mice with older grandfathers, suggesting that genetic and epigenetic alterations associated with APA are heritable [113].

Perinatal risk factors are also among the most-studied ASD risk factors and among the most difficult to determine and predict in advance. Two comprehensive meta-analyses examined 60 obstetric factors and found statistically significant associations between ASD risk and umbilical cord complications, injury or trauma at birth, multiple births, maternal hemorrhage, low birth weight, neonatal anemia, genital malformation, ABO or Rh blood group incompatibility and hyperbilirubinemia [114,115]. Another study has described an association between increased risk of autism and different factors, including caesarean section delivery, induced labor, management age less than 36 weeks and fetal distress [116].

Fetal exposure to sex steroids represents a potential risk factor for ASD. In fact, the fetal testosterone theory has been proposed to explain the higher ASD prevalence in males [8]. However, this hypothesis is controversial. In fact, while higher testosterone levels have been reported in ASD women, ASD males display testosterone levels similar to controls [117]. Baron-Cohen and colleagues have supposed that testosterone has effects on brain development during the prenatal masculinization window. The authors detected higher levels of sex steroids and cortisol in the amniotic fluid samples from male autistic patients compared with matched typically developing controls [118]. Recently, the same authors reported an association between fetal estrogen levels, important in synaptogenesis and corticogenesis, and autism risk [119]. Similarly, a study conducted on post-mortem brains detected reduced levels of estrogen receptor beta, aromatase and estrogen coactivators in the frontal gyrus of subjects with ASD compared to controls [120]. Moreover, several SNPs in genes encoding for proteins involved in sex steroid synthesis/transport have been associated with autistic traits [121].

The health condition of the mother has a major impact on the risk of ASD. It appears that maternal nutrition in pregnancy is of fundamental importance as it determines the nutrients available to support fetal growth [122]. Therefore, diets lacking specific nutrients can have adverse effects on fetal development. It has been shown that even short intervals between pregnancies can be harmful, since the body needs time, up to one year after childbirth, to recover acceptable levels of several essential substances [123].

Deficiencies of micronutrients, including vitamins and trace elements, have been associated with an increased risk of ASD. For instance, a Swedish study found that maternal vitamin D deficiency is associated with the risk of ASD with ID in the offspring [124]. Unbalanced levels of vitamins have also been detected in ASD children. Moreover, beneficial effects of vitamin supplementation have been observed in ASD patients [125]. Similarly, altered hair and/or blood concentrations of several trace elements, including chromium, magnesium and zinc, have been found in ASD patients compared with controls [126]. Association between maternal deficiency of microelements and ASD risk has also been reported. For example, iron deficiency, common in pregnant women, was associated with a five-fold increased risk of ASD, especially in the presence of other risk factors [127].

The involvement of altered trace elements concentrations on ASD phenotype is also supported by in vitro and animal studies. The effects on unbalanced metal levels on synapse formation and functionality have been evaluated on hippocampal cultured cells from rat brains, finding that the metal profile of autistic children led to down-regulation of crucial synaptic components, including Shank proteins and NMDA receptor subunits, and reduction of synaptic density. Interestingly, it was observed that zinc supplementation was able to revert the observed alterations [128]. The importance of zinc in ASD is supported by multiple pieces of evidence, including a strong association between low levels of this metal and ASD risk as well as a causative role of zinc deficiency in neuronal defects and development of ASD-related symptoms and “therapeutic” effects of zinc supplementation. In fact, low hair and serum levels of zinc and/or altered Zn/Cu ratio have been detected in children with ASD [129,130,131]. The effects of zinc deficiency on ASD have been observed in animal models. It has been shown that prenatal zinc deficiency alters social behavior in mice [132]. In vitro and in vivo studies have shown that zinc deficiency at the synaptic level leads to a decrease in ProSAP/Shank family members, a reduction in synaptic density and the development of ASD-related symptoms in mice [133]. In fact, other studies have shown that Zn^2+^ ions, highly abundant at PSD level, influence the recruitment of ProSAP1/Shank2 or ProSAP2/Shank3 for a correct formation and maturation of synapses [134]. The mechanism regulating the abundance of Zn at PSD levels has been hypothesized: Zn is released from pre-synaptic terminals and can translocate into post-synaptic neurons through Zn-permeable channels, including NMDA, and voltage-gated Ca^2+^ and AMPA channels [135]. Notably, a recent study has shown maternal zinc supplementation can prevent ASD-associated deficits in *Shank3* KO mouse models [136]. Recently, Shih et al. have elegantly demonstrated the impact of the crosstalk between genetic and environmental factors in the development of ASD. The authors have shown that KO of *Cttnbp2*, an actin cytoskeleton regulator, leads to a reduction in Zn concentration and expression levels of different synaptic proteins, consequently affecting dendritic spine formation and leading to the development of autism-like behaviors rescued by zinc supplementation [137]. The beneficial effects of zinc supplementation have been also observed in pregnant women, where an increase in zinc intake has been shown to reduce the risk of neural tube defects in the offspring [138].

The association between maternal obesity and risk of ASD in offspring is controversial. A Swedish study described a relationship between maternal BMI and ASD at population level; however, sibling analysis did not reveal any association between elevated maternal BMI and ASD risk [139]. On the other hand, a meta-analysis has shown a 28% and 36% increased risk of ASD in offspring born from overweight and obese mothers, respectively [140], even though there were relatively small numbers of ASD cases within the category of maternal underweight. However, it has also been shown that children born from underweight mothers are also at high risk of ASD [141]. Another study has shown that the combination of maternal obesity and maternal diabetes was associated with an increased risk of ASD and ID [142].

Maternal consumption of substances such as smoke, alcohol and medicines during pregnancy might be a potential risk factor as well. However, the association between smoking and alcohol-use with ASD is rather weak; in fact, two meta-analyses showed no evidence that smoking is a risk factor for ASD [143,144]. Moreover, cohort studies or case-control studies have examined the risk of ASD due to maternal alcohol consumption, indicating that mild to moderate consumption does not pose any risk [145,146,147,148].

The safety of medicines during pregnancy is very difficult to establish. In the ASD literature, antidepressant and anticonvulsant drugs have emerged as drugs of potential interest. An example is valproic acid, a drug that has been used to treat epilepsy or as a mood stabilizer in bipolar disorder. Its use during pregnancy leads to congenital malformations, developmental delay and cognitive malfunction [149,150]. Maternal use of selective serotonin uptake inhibitors has been associated with a 50% increase in ASD risk, although maternal psychiatric condition is a confounding factor [151].

An association between maternal diseases and ASD risk has been shown. A meta-analysis limited to case-control studies identified a 62% increased ASD risk among diabetic mothers compared to non-diabetic mothers [152], while a second study found a 74% increase in ASD risk for pregestational diabetes and 43% for gestational diabetes [153].

It has also been shown that maternal viral and bacterial infections are associated with ASD risk [154,155,156]. Two meta-analyses found that maternal autoimmune diseases are associated with an increased risk of ASD in offspring [157,158]. However, it is not the presence of viruses and bacteria per se to be associated with ASD development, but the immune response they cause, a conclusion supported by research identifying elevated inflammatory markers and antibodies in pregnant women with autistic children [159,160]. This hypothesis is supported by animal studies where maternal immune activation, induced by different immunogens, has been shown to induce post-natal brain dysfunction observable in a phenotype characteristic of ASD and other neurological disorders [161].

It is interesting to note that, although from different perspectives, much evidence points out the importance of maternal immune system condition during fetal development. Several studies have also reported an association between family history of autoimmune diseases and ASD [158,162,163]. It could be speculated that this link could be supported by maternal levels of zinc. In fact, our recent meta-analysis has shown that Zn concentrations in both serum and plasma levels of patients with autoimmunity are significantly lower compared to controls [164]. It is known that Zn plays an important role in the regulation of the immune system and, according to the studies provided above, it plays also an important role in neuronal tube formation.

Finally, exposure to toxic xenobiotics could represent another potential environmental risk factor. Substances such as brominated flame retardants could cause mitochondrial toxicity through a variety of mechanisms, leading to an altered energy balance in the brain [165], an important association with autism since mitochondrial dysfunction has been documented in patients with ASD [166]. Heavy metals can have a negative impact on many body functions by inducing neurological and behavioral damage. A meta-analysis of three case-control studies found a 60% increase in the risk of ASD due to exposure to high levels of inorganic mercury [167]. A recent case-control study found that exposure to organophosphates during pregnancy is associated with a 60% increase in the risk of ASD [168]. This category includes non-persistent organic pollutants, including phthalates and bisphenol A, and persistent organic pollutants, including DDT, PCB and PBDE. A research group found that three out of five studies on phthalate exposure showed a significant association between phthalate exposure and ASD risk [169]. Exposure to PCBs and PBDEs appears to alter calcium-related signal pathways, leading to alterations in dendritic growth and consequent abnormalities in neuronal connectivity, a key feature of ASD [170,171].

Table 2 summarizes several environmental factors associated with ASD described above.

## 5. Conclusions

Given the complexity of the etiology of autism and the increasing prevalence of new confirmed cases of ASD worldwide, there is an urgent need to find effective diagnostic methods and study as many risk factors as possible—not only genetic but also epigenetic and environmental ones—without neglecting also genetic/environmental interactions, where risk factors influence each other.

## Figures and Tables

**Table 1:** Several relevant ASD candidate genes.

Category	Gene Symbol	Gene Name	Alterations	Associated Syndromes
Chromatin regulators	*ANKRD11*	Ankyrin repeat domain 11	Mutations; copy number loss	KBG syndrome; Cornelia de Lange syndrome
	*ARID1B*	AT-rich interaction domain 1B	Mutations; copy number loss; copy number gain; translocation	Coffin–Siris syndrome
	*ASXL3*	ASXL Transcriptional Regulator 3	Mutations	Bainbridge-Ropers syndrome
	*ATRX*	ATRX Chromatin Remodeler	Mutations; copy number loss	
	*AUTS2*	Autism susceptibility candidate 2	Mutations; copy number loss; copy number gain; inversion; translocation	
	*CHD2*	Chromodomain helicase DNA binding protein 2	Mutations; copy number loss	Tourette syndrome
	*CHD7*	Chromodomain helicase DNA binding protein 7	Mutations; copy number loss; translocation	CHARGE syndrome
	*CHD8*	Chromodomain helicase DNA binding protein 8	Mutations; copy number loss; copy number gain; translocation	
	*CREBBP*	CREB-binding protein	Mutations; copy number loss	Rubinstein–Taybi syndrome, Menke-Hennekam syndrome 1, Tourette syndrome
	*EHMT1*	Euchromatic histone-lysine N-methyltransferase 1	Mutations; copy number loss; copy number gain; translocation	Kleefstra syndrome
	*MBD5*	Methyl-CpG binding domain protein 5	Mutations; copy number loss; copy number gain; inversion; translocation	2q23.1 microdeletion syndrome, Kleefstra syndrome
	*MECP2*	Methyl CpG binding protein 2	Mutations; copy number loss; copy number gain; promoter methylation	Rett syndrome, X-linked intellectual disability, MECP2 duplication syndrome
	*SETD5*	SET domain containing 5	Mutations; copy number loss	
Transcription factors/regulators	*ADNP*	Activity-dependent neuroprotector homeobox	Mutations; copy number loss	Helsmoortel-Van der Aa syndrome
	*FOXG1*	Forkhead box G1	Mutations; copy number loss; copy number gain; translocation	Rett syndrome, FOXG1 syndrome, West syndrome,
	*FOXP1*	Forkhead box P1	Mutations; copy number loss; inversion; translocation	
	*FOXP2*	Forkhead box P2	Mutations; copy number loss; translocation	FOXP2-related speech and language disorder
	*MED13L*	Mediator complex subunit 13-like	Mutations; copy number loss; copy number gain	
	*POGZ*	Pogo transposable element with ZNF domain	Mutations; copy number loss; copy number gain	White–Sutton syndrome
	*RAI1*	Retinoic Acid Induced 1	Mutations; copy number loss; copy number gain	Smith–Magenis syndrome, Potocki–Lupski syndrome
	*TBR1*	T-box, brain, 1	Mutations; copy number loss	
	*TCF4*	Transcription factor 4	Mutations; copy number loss; translocation	Pitt–Hopkins syndrome
	*ZBTB20*	Zinc finger and BTB domain containing 20	Mutations; copy number loss; translocation	3q13.31 microdeletion syndrome, Primrose syndrome,
mRNA binding and trafficking regulator	*FMR1* and its pathways	Fragile X mental retardation 1	Mutations; copy number loss	Fragile X syndrome, Fragile X-associated tremor/ataxia syndrome
Protein degradation	*UBE3A*	Ubiquitin protein ligase E3A	Mutations; copy number gain	Angelman syndrome
Cell growth/proliferation	*DYRK1A*	Dual-specificity tyrosine-(Y)-phosphorylation regulated kinase 1A	Mutations; copy number loss; inversion; translocation	
	*NF1*	Neurofibromin 1	Mutations; copy number loss	
	*PTEN* and its pathways	Phosphatase and tensin homolog	Mutations; copy number loss	Cowden syndrome, Macrocephaly/autism syndrome, PTEN hamartoma tumor syndrome
	*SYNGAP1*	Synaptic Ras GTPase activating protein 1	Mutations; copy number loss; translocation	
	*TSC1/TSC2*	Tuberous sclerosis 1/2	Mutations	
Protein modification	*CDKL5*	Cyclin-dependent kinase-like 5	Mutations; copy number loss; copy number gain translocation	Rett syndrome, Angelman syndrome

Prepared by the authors with data from gene.sfari.org (release 26 October 2020).

**Table 2:** Several environmental factors associated with ASD.

Factor	Evidence	References
Parental age	Association studies; meta-analyses; animal studies	[108,109,110,111,112,113]
Perinatal factors	Meta-analyses	[114,115,116]
Sex steroids	Association studies	[117,118,119,120,121]
Maternal nutrition	Association studies; meta-analyses; in vitro studies, animal studies	[123,124,125,126,127,128,129,130,131,132,133,134,136,137,138,139,140,141,142]
Fetal exposure to drugs, smoke, alcohol	Association studies; meta-analyses	[143,144,145,146,147,148,149,150,151]
Maternal diseases	Meta-analyses	[152,153]
Maternal infections	Association studies; meta-analyses; animal studies	[154,155,156,157,158,159,160,161]
Fetal exposure to toxic xenobiotics	Association studies; meta-analyses; in vitro studies; animal studies	[166,167,168,169,170,171]
